# Endotoxin Elimination in Patients with Septic Shock: An Observation Study

**DOI:** 10.1007/s00005-015-0348-8

**Published:** 2015-06-21

**Authors:** Barbara Adamik, Stanislaw Zielinski, Jakub Smiechowicz, Andrzej Kübler

**Affiliations:** Department of Anaesthesiology and Intensive Therapy, Wroclaw Medical University, Borowska 213, 50-556 Wroclaw, Poland

**Keywords:** Septic shock, Endotoxin elimination, Gram-negative infection, LPS measurement

## Abstract

To evaluate the effectiveness of endotoxin elimination with an adsorption column in patients with septic shock and endotoxemia. The elimination therapy was guided by a new bedside method of measuring endotoxin activity (EA). Intensive care unit (ICU) patients with septic shock and suspected Gram-negative infection were consecutively added to the study group within the first 24 h. Endotoxin elimination was performed using hemoperfusion with the Alteco LPS Adsorber. The primary endpoint was improvement in organ function within the first 24 h of treatment. A secondary objective was to assess the usefulness of a new method of measuring EA to help guide endotoxin elimination therapy. Out of 64 patients 18 had a high baseline EA [0.70 EA units (0.66–0.77)]. Those patients had endotoxin elimination treatment in addition to conventional medical therapy. At 24 h after endotoxin elimination, the EA had decreased to 0.56 EA units (0.43–0.77), (*p* = 0.005); MAP increased from 69 (62–80) to 80 mm Hg (68–88), (*p* = 0.002), and noradrenaline use decreased from 0.28 (0.15–0.80) to 0.1 μg/kg/min (0.00–0.70) at the same time (*p* = 0.04). The SOFA score had decreased from 11 (9–15) to 9 (7–14) points 24 h after endotoxin elimination (*p* = 0.01) with a median delta SOFA –2 points. Endotoxin elimination did not have a significant effect on the ICU length of stay or ICU mortality. Effective endotoxin elimination resulted in a significant improvement in hemodynamic parameters and of organ function. The application of the EA assay was useful for the bedside monitoring of endotoxemia in critically ill ICU patients.

## Introduction

Lipopolysaccharide (LPS) is a component of the outer membrane of Gram-negative bacteria and a well-known endotoxin which induces an inflammatory response (Marshall et al. 2004; Munford 2005; Murch et al. 2007). It is released during proliferation or lysis of bacterial cells. Gram-negative pathogens from the primary site of infection are the source of endotoxins in septic shock. Another cause of endotoxemia can be the transmucosal passage of either Gram-negative bacteria or just the LPS crossing from the intestines to sterile tissues. Endotoxins, either shed from the bacterial wall during an infection or after passing the intestinal barrier, are transported in the circulatory system in a complex with the LPS-binding protein. The LPS-cellular signaling pathway relies on the MD-2/TLR4 recognition complex and a membrane receptor CD14. The intracellular signaling cascade leads to the release of the inflammatory mediators that are typical for severe sepsis and septic shock (Burrell 1994). The key role of LPS in severe sepsis and septic shock was reported in several studies (Kojika et al. 2006; Monti et al. 2010; Opal et al. 1999; Silva et al. 2007), and an elevated endotoxin level was detected in patients with septic shock (Marshall et al. 2004; Murch et al. 2007). A high level of endotoxins is correlated with the degree of the cardiovascular failure (Monti et al. 2010; Murch et al. 2007), and with the acute physiology and chronic health evaluation (APACHE) II and sequential organ failure assessment (SOFA) scores (Marshall et al. 2004).

Extracorporeal blood purification therapies have been used to reduce endotoxin level in patients with sepsis (Davies and Cohen 2011). Recently, an endotoxin adsorber cartridge (Alteco LPS Adsorber, Alteco Medical AB, Lund, Sweden) has been introduced as a therapeutic intervention in septic shock. The cartridge is filled with polyethylene plates with a peptide adsorbing LPS with a high affinity. The aim of our study was to evaluate the effectiveness of endotoxin elimination with an Alteco LPS Adsorber in patients with septic shock and endotoxemia. Our primary endpoint was change in organ function within the first 24 h of treatment. The secondary objective was to assess the usefulness of a new bedside method of measuring endotoxin activity (EA) to help guide the LPS elimination therapy.

## Materials and Methods

### Patients

The study was conducted at the Department of Anesthesiology and Intensive Therapy, Wroclaw Medical University in Poland. The Ethics Committee of Wroclaw Medical University approved the study protocol. Endotoxin elimination with hemoperfusion described in our manuscript is accepted as a standard method of treatment in critically ill patients. The institutional review board granted exemption for this study, and the need for informed consent was waived. The setting was a 25-bed general intensive care unit (ICU) in a 996-bed tertiary-care university hospital. Adult patients with a diagnosis of septic shock, according to the definitions for sepsis and organ failure (American College of Chest Physicians/Society of Critical Care Medicine Consensus Conference: definitions for sepsis and organ failure and guidelines for the use of innovative therapies in sepsis 1992), and with a documented or suspected Gram-negative infection were consecutively added to the study group within the first 24 h after diagnosis. The exclusion criteria were: age less than 18 years, uncontrolled bleeding, pregnancy or terminal illness with no chance for meaningful recovery. Patients in whom the endotoxin elimination treatment could not be started within 24 h from the diagnosis of septic shock were excluded from the analysis. All patients in the study received a standard treatment for septic shock according to the Surviving Sepsis Campaign guidelines (Dellinger et al. 2008).

### Data Collection

The clinical status of the patients was assessed with the APACHE II score on admission to the ICU and with the SOFA score at the time of entry to the study group. Demographic data, microbiology results, length of ICU stay and survival were recorded. Hemodynamic and oxygenation variables were assessed: mean arterial pressure (MAP), heart rate, use of vasopressors, partial pressure of arterial oxygen (PaO_2_), arterial saturation, the oxygenation index. Routine parameters were also recorded such as lactate level, white blood cell (WBC) count, C-reactive protein (CRP) level, procalcitonin (PCT) level, creatinine and bilirubin level, coagulation parameters (APTT: activated partial thromboplastin time; PT: prothrombin time), and urine output. For patients who received endotoxin elimination treatment, all changes in parameters were recorded at 24 h after the first and second (if applied) session of endotoxin elimination. The ICU length of stay was counted until discharge from the ICU, death, or the 28th day after inclusion in the study.

### Endotoxin Elimination Method

Endotoxin elimination was performed using an Alteco LPS Adsorber column (Alteco Medical AB, Lund, Sweden). The adsorber is a class IIa medical device for extracorporeal removal of LPS from whole blood. This device contains polyethylene porous plates with a covalently bound peptide with a high affinity for endotoxins. When blood flows through the porous plates of the adsorber, the cationic peptide captures and eliminates negatively charged endotoxin molecules from the bloodstream. Hemoperfusion with the Alteco LPS Adsorber was performed using continuous renal replacement therapy equipment (multiFiltrate, Fresenius Medical Care, Bad Homburg, Germany) with a blood flow of 150 ml/h and unfractionated heparin anticoagulation. A double-lumen dialysis catheter was inserted into a central vein and used for venous access. The procedure was performed a maximum of two times. The first session of endotoxin elimination was completed within 120 min. The second session was performed 24 h after the end of the first session, also for 120 min. The decision to perform a second session was based on a persistently high EA level. For 12 patients, endotoxin elimination was performed simultaneously with renal replacement therapy.

### Endotoxin Activity Measurement

After diagnosis of septic shock, a blood sample (1 mL) was drawn from an intravenous catheter to a tube with an EDTA, as an anticoagulant agent, and EA was measured immediately. Endotoxemia was identified in whole blood samples with a commercially available, CE, IVD marked diagnostic endotoxin activity assay (EAA; Spectral Diagnostics Inc., Toronto, Canada). EAA is a rapid assay, based on the activation of neutrophils by endotoxins. A sample of the patient’s blood was incubated with the IgM antibody (raised against the lipid A of *E. coli J5*) and then stimulated with a zymosan. Oxygen radicals generated by primed neutrophils produced luminal chemiluminescence, and the signal was recorded with a luminometer (single tube luminometer, Smart Line TL, Berthold Detection Systems GmbH, Pforzheim, Germany). The results are quantitative, expressed in EA units (EAU), and they represent the mean value of duplicate analysis from each blood sample. The intra-assay coefficient of variation is 11 % and inter-assay coefficient of variation is 12 %. Endotoxin Activity Assay received FDA clearance in 2003 and European Regulatory Agency approval in 2004. Based on the manufacturer’s information, the EA level is considered to be low when it is <0.4 EAU, intermediate when it is between 0.4 and 0.59, and high when ≥0.6. The EA level was measured at the baseline for all patients diagnosed with septic shock. In patients with a high EA level (≥0.6 EAU) 2-h hemoperfusion with an LPS adsorber was performed in addition to standard treatment. Measurements of EA were repeated at 24 h after each session of LPS elimination. Patients with septic shock and EA < 0.6 EAU at the baseline received the full standard treatment, and EA measurements were done only at the baseline.

### Statistical Analysis

The data were analyzed with Statistica 10 (StatSoft, Inc. Tulsa, USA). The distribution of the variables was not normal based on a Shapiro–Wilk test. Therefore, statistical analysis of the data was performed using nonparametric techniques. Continuous variables are presented as medians with 25th and 75th percentiles. The baseline continuous variables were compared with the Mann–Whitney *U* test. Comparisons within a single group among different time points (baseline and 24 h) were performed by using a Wilcoxon rank sum test. The relationship between the EA and other parameters was assessed with a Spearman’s rank correlation test. Categorical variables were analyzed using a Chi-square test. Statistical significance was determined as *p* < 0.05.

## Results

### Baseline Characteristics

Sixty-four patients diagnosed with septic shock were treated in the ICU from February to December 2011. Two patients were excluded: one was terminally ill, with a “do not resuscitate” order, and the other had a source of infection that was impossible to remove surgically. A comparison of baseline parameters in patients with septic shock who received the standard treatment plus LPS elimination (*n* = 18) and those who received only the standard treatment (*n* = 44) is shown in Table 1.

**Table 1:** Baseline characteristics of the study group

	Patients with standard treatment and endotoxin elimination (*n* = 18)	Patients with standard treatment (*n* = 44)	*p*
LPS (EAU)	0.70 (0.66–0.77)	0.38 (0.25–0.42)	<0.0001
Age (years)	66 (38–75)	67 (59–76)	0.5
Gender (M/F)	15/3	27/18	0.1
APACHE II score	26 (17–30)	22 (16–27)	0.3
SOFA score	11 (9–15)	10 (8–13)	0.3
Noradrenaline, *n* (%)	18 (100)	44 (100)	0.1
Dobutamine, *n* (%)	2 (11)	5 (11)	0.8
Adrenaline, *n* (%)	7 (39)	15 (34)	0.6
Hydrocortisone therapy, *n* (%)	11 (61)	22 (50)	0.6
Total amount of fluids during the first 24 h (L)	7000 (4500–8600)	5700 (4600–7800)	0.6
Diagnosis on admission, *n* (%)			0.8
Intra-abdominal infection	13 (72)	30 (68)	
Pneumonia	3 (17)	10 (23)	
UTI	0	2 (5)	
Skin and soft tissue infection	1 (6)	2 (5)	
Meningitis	1 (6)	0	
ICU length of stay (days)	20 (15–30)	14 (8–31)	0.1
ICU 28 day mortality (%)	6 (33)	13 (30)	0.7

Continuous variables are presented as medians with 25th and 75th percentiles

*LPS* lipopolysaccharide, *apache* acute physiology and chronic health evaluation, *SOFA* sequential organ failure assessment, *CRP* C-reactive protein, *WBC* white blood cell count, *MAP* mean arterial pressure, *PaO*_*2*_ partial pressure of arterial oxygen, *PaO*_*2*_ */FiO*_*2*_ oxygenation index, *SaO*_*2*_ arterial saturation, *UTI* urinary tract infection, *ICU* intensive care unit

### Changes in Endotoxin Activity

**Fig. 1:**
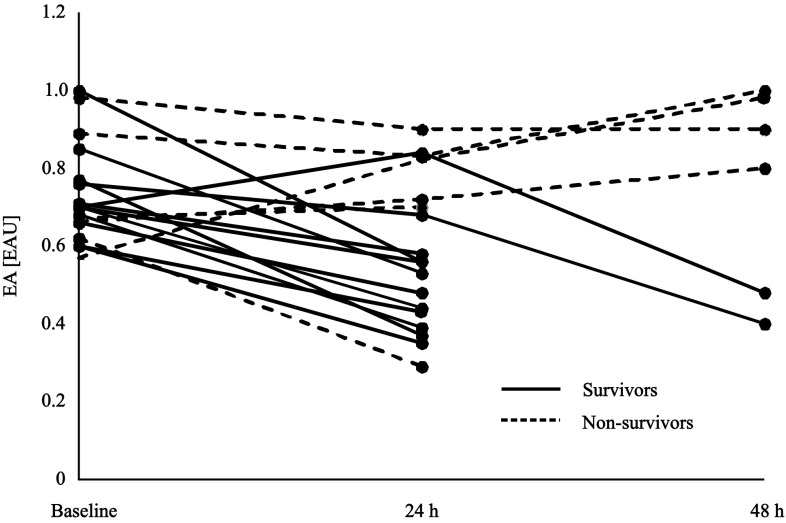
Endotoxin activity (EA) in the blood samples of patients with septic shock who received standard treatment plus LPS elimination, measured at the baseline (*n* = 18), at 24 h after the first session (*n* = 18), and 24 h after the second session (*n* = 6) of endotoxin elimination

The mean time from the diagnosis of septic shock to the start of endotoxin elimination treatment was 16 h (5–20). Low EA was detected in 25 (40 %) of the 62 patients with septic shock, intermediate in 19 (31 %) and high in 18 (29 %). In the standard treatment group, none of the measured EA exceeded the value of 0.6 EAU and median EA was 0.38 EAU (0.25–0.42). In contrast, for patients who received endotoxin elimination treatment, the baseline EA was significantly higher [0.70 EAU, (0.66–0.77)], (*p* < 0.0001), and all baseline values were ≥0.6 EAU. At 24 h after the first session of endotoxin elimination, the value of EA decreased to 0.56 EAU (0.43–0.77), (*p* = 0.005). A high EA level persisted in six patients and in those patients a second session of hemoperfusion was performed. This resulted in a decrease in EA to an intermediate level in two patients. Four patients continued to have a high endotoxin level [0.94 EAU, (0.85–0.99)], despite the second session of hemoperfusion with the LPS adsorber (Fig. 1). All of those patients died as a result of a multiple organ failure. The detailed reasons of death were as follows: (1) fulminant streptococcal septic shock, (2) liver necrosis, (3) metastatic neoplasm, and (4) multiple inoperable intra-abdominal abscesses.

### Hemodynamic and Oxygenation Variables

At baseline, all patients were receiving noradrenaline (NR), 11 % of patients were receiving dobutamine, and 35 % adrenaline (Table 1). At baseline, there were no significant differences in the MAP and vasopressor requirements between the two groups, despite the differences in the endotoxin levels (Table 1). For 18 patients who received endotoxin elimination treatment, a significant increase in the value of MAP was observed at 24 h after the first session (*p* = 0.002), and the noradrenaline use decreased at the same time (*p* = 0.04) (Table 2). Six patients required a second session of elimination. At 24 h after the second session, the MAP had increased to a mean 88 mm Hg and NR use decreased to a mean 0.28 µg/kg/min for two patients whose endotoxin level was successfully lowered to the intermediate level; in four cases, with ineffective endotoxin elimination, the MAP did not change significantly (70 mm Hg, (60–75) and NR use increased to 1.04 µg/kg/min (0.72–1.41). There were no significant changes in hemodynamic parameters observed in the standard treatment group after 24 h, compared to the baseline values: MAP was 66 mm Hg (55–70) and the NR dose was 0.36 µg/kg/min (0.09–1.4) (Table 1).

**Table 2:** Changes in the parameters of patients receiving endotoxin elimination therapy and in the standard treatment group

	Patients with standard treatment and endotoxin elimination			Patients with standard treatment		
	Baseline	After 24 h	*p*	Baseline	After 24 h	*p*
LPS (EAU)	0.70 (0.66–0.77)	0.56 (0.43–0.77)	0.005	0.38 (0.25–0.42)	–	–
Lactate (mmol/L)	3.03 (1.9–5.2)	2.29 (1.2–3.8)	0.03	2.5 (1.6–4.3)	3.4 (1.5–4.6)	n.s.
Noradrenaline (µg/kg/min)	0.28 (0.15–0.80)	0.10 (0.00–0.70)	0.04	0.22 (0.15–0.44)	0.36 (0.09–1.40)	n.s.
MAP (mm Hg)	69 (62–80)	80 (68–88)	0.002	67 (60–73)	66 (55–70)	n.s.
PaO_2_ (mm Hg)	97.4 (76.0–112.0)	90.0 (83.4–112.0)	n.s.	100.2 (87.7–110.5)	92.5 (89.0–98.0)	n.s.
SaO_2_ (%)	97.0 (94–98.5)	97.8 (95.3–98.6)	n.s.	97.4 (96.2–97.9)	97.6 (97.2–98.0)	n.s.
PaO_2_/FiO_2_	237 (124–270)	250 (180–315)	n.s.	260 (164–328)	225 (200–265)	n.s.
Urine output (ml/kg/h)	0.32 (0.0–1.0)	0.00 (0.0–0.6)	n.s.	0.8 (0.0–1.5)	0.5 (0.0–1.5)	n.s.
Creatinine (mg/dl)	1.7 (1.1–2.4)	1.7 (1.0–2.7)	n.s.	1.5 (1.1–3.1)	1.6 (1.0–2.5)	n.s.
CRP (mg/l)	189 (134–227)	197 (109–249)	n.s.	165 (100–247)	166 (89–215)	n.s.
Procalcitonin (ng/ml)	25.5 (5.6–92.4)	21.8 (2.6–91.2)	n.s.	7.4 (2.5–41.3)	13.2 (3.5–27.2)	n.s.
Bilirubin (mg/dl)	1.4 (0.8–3.1)	1.3 (0.6–2.8)	n.s.	1.2 (0.6–2.6)	1.2 (0.8–2.5)	n.s.
WBC (10^3^/µl)	15.4 (7.9–23.2)	14.9 (11.8–19.6)	n.s.	20.1 (11.3–31.0)	16.2 (13.1–30.5)	n.s.
Platelets (10^3^/µl)	136.5 (46.0–258.0)	111.0 (35.0–189.0)	0.002	128 (82–250)	129.5 (61.5–249.5)	n.s.
APTT (sec)	53.1 (47.3–63.9)	55.4 (45.7–74.5)	n.s.	45.8 (37.0–54.6)	43.4 (38.7–50.0)	n.s.
PT (%)	77.9 (64.2–88.5)	80 (60.8–88.0)	n.s.	75.8 (69.8–85.0)	76.7 (68–86)	n.s.
D-dimers (µg/ml)	8.1 (5.2–10.6)	13.0 (5.0–17.2)	n.s.	5.5 (4.0–11.0)	6.4 (3.7–9.4)	n.s.

*p* value represents statistical significance with reference to the baseline values; continuous variables are presented as medians with 25th and 75th percentiles

*LPS* lipopolysaccharide, *SOFA* sequential organ failure assessment, *MAP* mean arterial pressure, *PaO*_*2*_ partial pressure of arterial oxygen, *PaO*_*2*_ */FiO*_*2*_ oxygenation index, *SaO*_*2*_ arterial saturation, *CRP* C-reactive protein, *WBC* white blood cell count, *APTT* activated partial thromboplastin time, *PT* prothrombin time, *n.s.* non-significant

Endotoxin elimination did not have a significant impact on changes in the oxygenation parameters: PO_2_, SaO_2_ and PaO_2_/FiO_2_ (Table 2). At 24 h after the second session of elimination no significant changes in oxygenation parameters were observed (data not shown).

### Organ Function

There was a significant decrease in the SOFA score from the baseline to 24 h after endotoxin elimination (Table 3) with a delta SOFA score –2.0 points (–3–0.5). The improvement in the cardiovascular system was responsible for the change in the SOFA score (*p* = 0.007). No change in the SOFA score was observed in the conventional therapy group. The lactate level had also decreased significantly (*p* = 0.03) 24 h after endotoxin elimination; this was not observed in the conventional therapy group (Table 2). Out of 62 cases, 32 patients (52 %) had acute kidney injury at the time of entry to the study and required renal replacement therapy. We did not observe any significant effect on renal function. PCT and CRP levels were elevated in all patients and no marked changes in these parameters were recorded from the baseline to 24 h after endotoxin elimination (Table 2). A weak positive correlation between the PCT level and EA (*R* = 0.3, *p* = 0.04) was recorded. The PCT level was much higher in patients with endotoxemia, compared to the standard treatment group, but the difference was not statistically significant. There was no correlation of EA with CRP, noradrenaline dose, or the SOFA score. For 18 patients who received endotoxin elimination treatment, leukopenia was present in 16 %, leukocytosis in 67 and 17 % had a WBC within normal range at baseline; no significant changes in WBC were observed 24 h after endotoxin elimination. Platelet count dropped significantly from 136.5 to 111.0 × 10^3^/µl at 24 h. The decrease in platelet count was more pronounced in the 12 patients who had endotoxin elimination performed simultaneously with renal replacement therapy, although there were no noted problems with hemoperfusion bleeding either during or after the procedure. No significant changes in APTT, PT and D-dimers were noticed (Table 2). The ICU length of stay and 28-day ICU mortality was similar among the studied patients (Table 1). The standardized mortality ratio was 1.14 in patients with endotoxemia and 1.01 in the standard treatment group.

**Table 3:** Organ dysfunction indicated by SOFA score in patients receiving endotoxin elimination therapy and in the standard treatment group

	Patients with standard treatment and endotoxin elimination			Patients with standard treatment		
	Baseline	After 24 h	*p*	Baseline	After 24 h	*p*
Total SOFA points	11 (9–15)	9 (7–14)	0.01	10 (8–13)	12 (9–14)	n.s.
Respiratory	2 (2–3)	2 (1–3)	n.s.	2 (2–3)	2 (2–3)	n.s.
Hematologic	1 (0–3)	1 (0–3)	n.s.	1 (0–2)	1 (0–2)	n.s.
Cardiovascular	4 (4–4)	3 (0–4)	0.007	4 (4–4)	4 (4–4)	n.s.
Hepatic	1 (0–2)	1 (0–2)	n.s.	1 (0–2)	1 (0–2)	n.s.
Renal	3 (0–4)	3 (0–4)	n.s.	1 (0–4)	1 (1–4)	n.s.
Central nervous system	0 (0–2)	0 (0–2)	n.s.	0 (0–2)	0 (0–2)	n.s.

*p* value represents statistical significance with reference to the baseline values; continuous variables are presented as medians with 25th and 75th percentiles

### Pathogen Identification

We observed that there was a predominance of Gram-negative pathogens as the source of infection leading to septic shock (Table 4). Out of 62 cases, Gram-negative bacteria was detected in 44 (71 %) patients, Gram-positive in 15 (24 %), fungi in 2 (3 %), and other pathogens in 2 patients (4.5 %, *Mycoplasma pneumoniae*). There were no significant differences in the type of identified pathogen between patients subjected to endotoxin elimination treatment and those who received a standard treatment for septic shock. The baseline EA level was 0.44 EAU (0.34–0.68) in patients with a Gram-negative infection and 0.41 EAU (0.32–0.58) in those with Gram-positive, fungal or other infections, but the observed difference was not statistically significant (*p* = 0.23). Pathogens in the blood were detected in only one-fifth of the 62 cases and no difference between the groups was observed.

**Table 4:** The identification of pathogens in the blood and other specimens collected within the first 24 h after a diagnosis of septic shock

	G (−)	G (+)	Fungi/other	*p*
Patients with standard treatment and endotoxin elimination, *n* (%)	14 (78)	3 (17)	1 (6)/0	0.0001
Patients with standard treatment, *n* (%)	30 (68)	12 (27)	1 (2)/2 (4.5)	0.006
Total, *n* (%)	44 (71)	15 (24)	2 (3)/2 (4.5)	0.0001

*p* value for comparison of all three categories of pathogens

*G* (−) gram-negative bacteria, *G* (+) gram-positive bacteria

## Discussion

The results of our study show that endotoxin elimination was effective in patients with septic shock and was associated with improvement in organ function, indicated by the SOFA score, blood pressure, and vasopressor requirements. In most studies to date, adsorbers containing polymyxin B immobilized to polystyrene fibers were used to adsorb circulating LPS, thereby neutralizing the effects of this endotoxin (Nemoto et al. 2001; Tani et al. 2010). However, in the majority of studies the effectiveness of the endotoxin elimination therapy was not supported by monitoring the endotoxin blood level. To guide endotoxin elimination therapy, we used a new bedside method of measuring EA. For LPS neutralization, we utilized a new endotoxin adsorber cartridge, an Alteco LPS adsorber, with a specific, tailor-made cationic polypeptide bound to polyethylene discs. The polypeptide binds with a high affinity to lipid A, the negatively charged fragment of bacterial LPS. The major difference between the polymyxin B cartridge and the Alteco LPS adsorber column is that the latter does not have antibiotics immobilized to the fibers. The procedure of endotoxin elimination is similar for both devices, i.e. 2-h direct hemoperfusion with a blood flow rate of 150 ± 50 mL/min for the Alteco LPS adsorber column and 80–120 mL/min for the polymyxin B cartridge.

Clinical experience with the Alteco LPS Adsorber column is very limited, and so far only a few studies have been published (Ala-Kokko et al. 2011; Blomquist et al. 2009; Kulabukhov 2008; Yaroustovsky et al. 2009). Our study noted a significant decrease in the EA level within 24 h after the first hemoperfusion. Ala-Kokko reported a similar decrease in EA in a group of patients with septic shock within 24 h after treatment with an adsorber (Ala-Kokko et al. 2011). Another study showed a decrease of 76 % in the LPS level in a group of patients with Gram-negative nosocomial pneumonia within 48 h after hemoperfusion (Yaroustovsky et al. 2009).

The effective elimination of endotoxin was associated with a significant increase in the mean arterial pressure noted at 24 h after endotoxin elimination, even though the dose of noradrenaline had been significantly reduced at the same time. Therefore, based on these preliminary results, one of the reasons why endotoxin elimination therapy may be beneficial in septic patients is from an improvement in hypotension. In the study done by Danner, patients with septic shock and endotoxemia had low systemic vascular resistance and depressed ejection fraction compared to patients without endotoxemia (Danner et al. 1991). Several studies reported the beneficial effect of endotoxin elimination with either an Alteco LPS Adsorber or polymyxin B column. In the EUPHAS trial, the MAP increased and vasopressor requirements decreased significantly 72 h after hemoperfusion (Cruz et al. 2009). In another study, a marked reduction in the noradrenaline infusion rate at 24 h after treatment was observed; however, it was not accompanied by an increase in the MAP (Ala-Kokko et al. 2011).

In the present study, all patients had elevated PCT and CRP levels, 83 % had either leukopenia or leukocytosis, indicating the induction of systemic inflammation in the course of septic shock. The baseline PCT level was over three times higher in the group with endotoxemia than in patients without elevated LPS activity. The difference observed between groups at baseline indicates that endotoxemia is accompanied with high PCT, even though the result was not statistically significant. PCT decreased in response to the endotoxin elimination treatment and increased almost twice as much in patients who had had standard treatment of septic shock. These changes were not statistically significant; however, there was an observed tendency toward improvement in patient condition at 24 h after endotoxin elimination. Several studies have shown a correlation between endotoxemia and PCT level. In patients with septic complications after cardiac surgery, a significant correlation between EA and PCT level was noted (Yaroustovsky et al. 2013). In another study, low EA was associated with only a slight rise in PCT level; however, when EA was ≥0.6 EAU, procalcitonin increased over four times (Yaguchi et al. 2012). Neither CRP nor WBC could accurately predict the response to treatment at 24 h after endotoxin elimination, despite a significant decrease in LPS activity and improvement in the hemodynamic status of patients. This finding is in agreement with the results of another study that evaluated the usefulness of CRP and PCT as clinical and biological markers in septic shock (Claeys et al. 2002); no changes in CRP and PCT were observed at 24 and 48 h of treatment vs. baseline. In addition, PCT and CRP levels did not differ significantly between survivors and non-survivors. Clearly, the association between PCT and endotoxemia in septic shock is not fully understood, and additional studies with a bigger sample size are needed.

The significant decrease in platelet count after LPS elimination was similar to that reported in several previous studies (Ala-Kokko et al. 2011; Ikeda 2002), but there were no bleeding events observed in our study either during or after the procedure. The drop in the platelet count was more pronounced in patients who had endotoxin elimination therapy performed simultaneously with renal replacement therapy. This suggests that the decrease in platelet count could have been due to hemodilution; however, the direct interaction of platelets with the hemoperfusion devices cannot be excluded. Although no bleeding problems were reported either during or after the studied procedure, special attention should be given to patients who have low platelet count at baseline.

Several studies have reported that patients with an elevated endotoxin level have an increased risk of ICU mortality (Danner et al. 1991; Marshall et al. 2004; Opal et al. 1999; Yaguchi et al. 2012). The presence of endotoxemia in a group of patients with septic shock and positive blood culture was associated with 39 % mortality in contrast to 7 % in those without endotoxemia (Danner et al. 1991). In another study, which evaluated patients with suspected sepsis, ICU mortality was 37 % when EA was >0.6 EAU and 17.3 % when EA was low (Yaguchi et al. 2012). In our study, there was no difference in the mortality rate in patients with a high or low endotoxin level at baseline, although previously published results would have indicated that patients with a higher endotoxin level would have higher mortality. Since the presence of endotoxemia identifies a population of patients with an increased risk of dying, the fact that there was no difference in mortality observed in our study might suggest the beneficial effect of endotoxin elimination treatment on survival. We have to acknowledge that the number of cases included in the project did not allow for a comprehensive analysis of the outcome, and any conclusions based on the results of such a small population would be overstated.

Gram-negative bacteria were the most often isolated pathogens in patients with septic shock (71 % of patients). Not all patients with documented Gram-negative infection had endotoxemia. Only one-third of them had a high EA level which required elimination treatment, and in two-thirds of the patients, despite the confirmed identification of a Gram-negative infection, the EA level was <0.6 EAU. For those patients, endotoxin elimination treatment was not applied. One-third of the septic shock patients had a documented Gram-positive bacteria, fungi or mycoplasma. In some of these cases, the high activity of endotoxins was detected and the elimination procedure was performed, even though a Gram-negative pathogen could not be detected. An infection with Gram-negative bacteria is usually associated with a high level of endotoxemia (Marshall et al. 2004). However, studies on the prevalence of endotoxemia in critically ill patients have shown that LPS is detected much more often than a microbiologically proven, Gram-negative infection (Danner et al. 1991; Marshall et al. 2004; Opal et al. 1999). In critically ill patients endotoxemia can be the result of changes in permeability in the gastrointestinal tract and the subsequent translocation of LPS and Gram-negative bacteria to the bloodstream. Therefore, endotoxemia can be detected also in septic patients with a microbiologically proven, Gram-positive or fungal infection. This finding was reported previously (Danner et al. 1991; Marshall et al. 2004; Murch et al. 2007; Vincent et al. 2005), suggesting that the injured intestinal mucosal barrier may be the reservoir of Gram-negative pathogens and endotoxins.

The current clinical challenge is to identify patients for whom endotoxin elimination treatment would be the most beneficial. In a study by Vincent et al. (2005), the population was limited to surgical patients with severe sepsis or septic shock, with a presumed Gram-negative infection originating from the abdominal cavity. In another clinical study (EUPHAS), the population was narrowed to patients with severe sepsis or septic shock caused by an intra-abdominal cavity infection (Cruz et al. 2009). There was a high probability of endotoxemia in these two narrowly defined populations. In our research, the study population was diverse, both medical and surgical patients with a documented or suspected Gram-negative infection. Endotoxemia had to be either confirmed or ruled out in each case. The clinical effect of LPS elimination in septic patients was previously reported; however, projects supported by LPS measurements for controlling the effectiveness of the treatment are rare. The EA assay which was applied in this study, allowed rapid and specific detection of the lipid A epitope of the lipopolysaccharide molecule. This quantitative assay has been widely used to detect endotoxemia in human blood samples (Ala-Kokko et al. 2011; Marshall et al. 2004; Romaschin et al. 2012; Yaguchi et al. 2012). The bedside measurements of the EA level in whole blood were helpful in making a decision about when to start endotoxin elimination. Therefore, patients with high EA who were suspected of having a Gram-negative infection from a source other than the abdominal cavity were also included in the study. The decision about a second session of endotoxin elimination was made based on the results of the EA. Less than half of the patients required a second elimination treatment. In 4 of 18 cases, LPS elimination was ineffective and the EA had risen despite treatment. The reason could have been associated with inadequate antibiotic treatment or a surgically inoperable source of infection.

There are no strict recommendations regarding how many times the endotoxin elimination therapy should be repeated for one patient. The procedure with either an Alteco LPS adsorber or polymyxin B cartridge was performed for 2 h once (Ala-Kokko et al. 2011; Blomquist et al. 2009; Nemoto et al. 2001; Vincent et al. 2005), twice (Cruz et al. 2009; Nakamura et al. 2005) or three (Tani et al. 2001) times. We performed the procedure a maximum of two times, depending on the patient’s clinical response and EA. In our opinion, measuring EA with an EAA was essential for both identifying patients who needed endotoxin elimination and monitoring the effect of the intervention. We chose the EA level ≥0.6 EAU based on the results from previously published studies (Marshall et al. 2004) and on the manufacturer’s recommendations. A low level of EA was previously noted in a majority of healthy volunteers (median: 0.26 EAU), and all healthy volunteers had an EA level below 0.6 EAU (Marshall et al. 2004). The inclusion criterion of EA ≥ 0.6 EAU has been used in ongoing clinical trials to assess the efficacy of endotoxin elimination in septic shock—the EUPHRATES trial and EUPHAS 2 trial.

It should be emphasized that the LPS level is regulated through mechanisms for LPS detoxification. Those mechanisms include: LPS uptake into the liver, LPS-binding to prevent the engaging of TLR4, the modulation of the target cell response, and LPS enzymatic degradation. Detoxification mechanisms depend on many factors related to the type and dose of LPS, the clinical status of the patient, and treatment methods, and large individual variations have been seen (Munford 2005). Any type of LPS elimination method should be considered as a support to the natural mechanisms of LPS detoxification. We acknowledge that our study had some limitations. Firstly, it was an observational study, there was no randomized control group with EA level ≥0.6 EAU, and the outcome of the patients was not established as the key indicator. Secondly, not all patients had cardiac output monitoring. For this reason, we could not provide detailed hemodynamic data. Thirdly, patients who received the standard treatment were evaluated only for baseline EA. However, our primary interest was to study the effectiveness of the endotoxin adsorption method, not the prevalence of endotoxemia in septic patients.

In conclusion, this study demonstrated that the use of an Alteco LPS Adsorber resulted in the effective elimination of endotoxins from the blood of septic shock patients. The LPS elimination by hemoperfusion supported the natural mechanisms for LPS-binding. The therapy was associated with an increase in blood pressure and a reduction of vasopressor requirements. The decrease in the SOFA score indicated significant improvement in organ function. Patients diagnosed with septic shock and with a suspected Gram-negative infection did not always have a high endotoxin level. A lack of endotoxin monitoring might explain the unsuccessful results of earlier studies aimed at neutralizing endotoxins in septic patients (Angus et al. 2000; McCloskey et al. 1994). Our study showed the usefulness of the EAA in qualifying patients for endotoxin elimination treatment. The application of the assay identified patients who might benefit from the therapy. Further projects, involving a larger group of patients, are needed to define when endotoxin elimination treatment should start and how many times it should be repeated.

In summary:

1. Hemoperfusion with an LPS adsorber added to the standard treatment of septic shock improves organ function, as indicated by the reduction of vasopressor requirements and elevation of blood pressure.
2. A high level of EA, despite endotoxin elimination therapy, may indicate poor control over the source of infection, suggesting the need for more active diagnostic and treatment strategies.
3. Our study demonstrates the clinical significance of measuring EA when selecting septic patients for endotoxin elimination treatment. Endotoxin activity measurement is available as a simple and quick bedside method that aids in the diagnostics of severe sepsis.

## References

[CR1] (1992) American College of Chest Physicians/Society of Critical Care Medicine Consensus Conference: definitions for sepsis and organ failure and guidelines for the use of innovative therapies in sepsis. Crit Care Med 20:864–874 1597042

[CR2] Ala-Kokko TI, Laurila J, Koskenkari J (2011). A new endotoxin adsorber in septic shock: observational case series. Blood Purif.

[CR3] Angus DC, Birmingham MC, Balk RA (2000). E5 murine monoclonal antiendotoxin antibody in gram-negative sepsis: a randomized controlled trial. E5 Study Investigators. JAMA.

[CR4] Blomquist S, Gustafsson V, Manolopoulos T (2009). Clinical experience with a novel endotoxin adsortion device in patients undergoing cardiac surgery. Perfusion.

[CR5] Burrell R (1994). Human responses to bacterial endotoxin. Circ Shock.

[CR6] Claeys R, Vinken S, Spapen H (2002). Plasma procalcitonin and C-reactive protein in acute septic shock: clinical and biological correlates. Crit Care Med.

[CR7] Cruz DN, Antonelli M, Fumagalli R (2009). Early use of polymyxin B hemoperfusion in abdominal septic shock: the EUPHAS randomized controlled trial. JAMA.

[CR8] Danner RL, Elin RJ, Hosseini JM (1991). Endotoxemia in human septic shock. Chest.

[CR9] Davies B, Cohen J (2011). Endotoxin removal devices for treatment of sepsis and septic shock. Lancet Infect Dis.

[CR10] Dellinger RP, Levy MM, Carlet JM (2008). Surviving Sepsis Campaign: international guidelines for management of severe sepsis and septic shock: 2008. Intensive Care Med.

[CR11] Ikeda T (2002). Hemoadsorption in critical care. Ther Apher.

[CR12] Kojika M, Sato N, Yaegashi Y (2006). Endotoxin adsorption therapy for septic shock using polymyxin B-immobilized fibers (PMX): evaluation by high-sensitivity endotoxin assay and measurement of the cytokine production capacity. Ther Apher Dial.

[CR13] Kulabukhov VV (2008). Use of an endotoxin adsorber in the treatment of severe abdominal sepsis. Acta Anaesthesiol Scand.

[CR14] Marshall JC, Foster D, Vincent JL (2004). Diagnostic and prognostic implications of endotoxemia in critical illness: results of the MEDIC Study. J Infect Dis.

[CR15] McCloskey RV, Straube RC, Sanders C (1994). Treatment of septic shock with human monoclonal antibody HA-1A. A randomized, double-blind, placebo-controlled trial. CHESS Trial Study Group. Ann Intern Med.

[CR16] Monti G, Bottiroli M, Pizzilli G (2010). Endotoxin activity level and septic shock: a possible role for specific anti-endotoxin therapy?. Contrib Nephrol.

[CR17] Munford RS (2005). Detoxifying endotoxin: time, place and person. J Endotoxin Res.

[CR18] Murch O, Collin M, Hinds CJ (2007). Lipoproteins in inflammation and sepsis. I. Basic science. Intensive Care Med.

[CR19] Nakamura T, Kawagoe Y, Sukuzi T (2005). Changes in plasma interleukin-18 by direct hemoperfusion with polymyxin B-immobilized fiber in patients with septic shock. Blood Purif.

[CR20] Nemoto H, Nakamoto H, Okada H (2001). Newly developed immobilized polymyxin B fibers improve the survival of patients with sepsis. Blood Purif.

[CR21] Opal SM, Scannon PJ, Vincent JL (1999). Relationship between plasma levels of lipopolysaccharide (LPS) and LPS-binding protein in patients with severe sepsis and septic shock. J Infect Dis.

[CR22] Romaschin AD, Klein DJ, Marshall JC (2012). Bench-to-bedside review: clinical experience with the endotoxin activity assay. Crit Care.

[CR23] Silva E, Arcaroli J, He Q (2007). HMGB1 and LPS induce distinct patterns of gene expression and activation in neutrophils from patients with sepsis-induced acute lung injury. Intensive Care Med.

[CR24] Tani T, Hanasawa K, Kodama M (2001). Correlation between plasma endotoxin, plasma cytokines, and plasminogen activator inhibitor-1 in septic patients. World J Surg.

[CR25] Tani T, Shoji H, Guadagni G (2010). Extracorporeal removal of endotoxin: the polymyxin B-immobilized fiber cartridge. Contrib Nephrol.

[CR26] Vincent JL, Laterre PF, Cohen J (2005). A pilot-controlled study of a polymyxin B-immobilized hemoperfusion cartridge in patients with severe sepsis secondary to intra-abdominal infection. Shock.

[CR27] Yaguchi A, Yuzawa J, Klein DJ (2012). Combining intermediate levels of the Endotoxin Activity Assay (EAA) with other biomarkers in the assessment of patients with sepsis: results of an observational study. Crit Care.

[CR28] Yaroustovsky M, Abramyan Z, Popok Z (2009). Preliminary report regarding the use of selective sorbents in complex cardiac surgery patients with extensive sepsis and prolonged intensive care stay. Blood Purif.

[CR29] Yaroustovsky M, Plyushch M, Popov D (2013). Prognostic value of endotoxin activity assay in patients with severe sepsis after cardiac surgery. J Inflamm.

